# A Mobile Phone App for the Self-Management of Pediatric Concussion: Development and Usability Testing

**DOI:** 10.2196/12135

**Published:** 2019-05-31

**Authors:** Harminder Sandhu, Katherine Wilson, Nick Reed, Alex Mihailidis

**Affiliations:** 1 Institute of Biomaterials & Biomedical Engineering University of Toronto Toronto, ON Canada; 2 Concussion Centre Bloorview Research Institute Holland Bloorview Kids Rehabilitation Hospital Toronto, ON Canada; 3 Rehabilitation Sciences Institute Faculty of Medicine University of Toronto Toronto, ON Canada; 4 Department of Occupational Science and Occupational Therapy Faculty of Medicine University of Toronto Toronto, ON Canada; 5 Toronto Rehabilitation Institute University Health Network University of Toronto Toronto, ON Canada

**Keywords:** brain concussion, safety, pediatrics, youth, children, self-management, mild traumatic brain injury, mobile apps, mobile health

## Abstract

**Background:**

Concussion is a common injury among Canadian children and adolescents that leads to a range of neurobehavioral deficits. However, noticeable gaps continue to exist in the management of pediatric concussion, with poor health outcomes associated with the inadequate application of best practice guidelines.

**Objective:**

The aim of this study was to describe the development and assess the usability of a mobile phone app to aid youth in the self-management of concussion. A secondary objective was to assess the usefulness of the app.

**Methods:**

An agile user-centered design approach was used to develop the technology, followed by a formative lab-based usability study for assessment and improvement proposals. Youths aged 10 to 18 years with a history of concussion and health care professionals involved in concussion management were recruited. This study included participants performing 12 tasks with the mobile phone app while using the *think aloud* protocol and the administration of the System Usability Scale (SUS), posttest questionnaire, and a semistructured interview.

**Results:**

A mobile phone app prototype called *NeuroCare*, an easily accessible pediatric concussion management intervention that provides easy access to expert-informed concussion management strategies and helps guide youth in self-managing and tracking their concussion recovery, was developed. A total of 7 youths aged between 10 and 18 years with a history of concussion and 7 health care professionals were recruited. The mean SUS score was 81.9, mean task success rates were greater than 90% for 92% (11/12) of the tasks, 92% (11/12) of tasks had a total error frequency of less than 11 errors, and mean task completion times were less than 2 min for 100% of the tasks.

**Conclusions:**

Results suggest that participants rated this app as highly usable, acceptable to users, and that it may be useful in helping youth self-manage concussion.

## Introduction

### Background

Concussion is a common injury [1,2] (200 per 100,000 [3]) among Canadian children and adolescents that leads to a range of neurobehavioral deficits including combinations of somatic, physical, cognitive, and emotional and behavioral symptoms [4]. These postconcussion symptoms can have a significant impact on the functional participation of youth in daily activities, such as sports, school, as well as family and social activities [3,5,6]. There is a lack of evidence-based interventions for the management of pediatric concussion [6,7], but consistent application of best practice guidelines may help reduce the impact of concussion and persistent postconcussion symptoms [8]. However, noticeable gaps continue to exist in concussion management with inadequate application of best practice guidelines, and there is growing evidence demonstrating both knowledge and practice gaps in concussion management [8-11]. Consequently, individuals may receive inconsistent and incomplete messages regarding the best strategies to manage concussion, which could lead to poor health outcomes.

### Concussion & You

*Concussion & You* is an evidence-informed self-management education program for concussed youth and their families [12]. It features a concussion curriculum based on best evidence and expert opinion and is integrated within a self-management framework. *Concussion & You* aims to provide evidence-informed best practice guidance regarding concussion recovery throughout the entire recovery process and enable participants to build an idiosyncratic concussion recovery toolkit using the practical concussion management strategies provided by the program for the management of return to school and play, sleep, nutrition, relaxation, and energy conservation that the youth can access throughout their recovery [12,13]. The feasibility of this program was validated in a pilot study that led to an increase in patients’ knowledge regarding concussion and concussion management strategies after intervention [12]. Youths and their families are able to implement strategies into their daily routines with the use of supplied daily planners, activity logs, and postconcussion symptom scales; results from the postsession survey indicated that these tangible tools positively affect participants’ recovery [12]. This program addresses many gaps in concussion management, which can significantly improve the quality of life and outcomes of concussed youth, but it currently relies on in-person interaction and only at 1 time point with no additional follow-up or support.

### Mobile Health

Many clinical researchers have begun harnessing technology to develop innovative approaches that hold great promise for enhancing the accessibility and quality of care [14,15]. Mobile health (mHealth) technologies, such as mobile phones, are well suited to serve as platforms for the self-management of health conditions as they are ubiquitous, have great computational capabilities, and are commonly carried on the person [13,16]. In addition, mHealth technologies can facilitate access to self-monitoring resources, time-sensitive health information, prompts, reminders, and personalized self-management tools in real time [13,16]. Mobile phones are ubiquitous in the lives of youth [17,18], so interventions using mobile technology may provide important and innovative opportunities for engaging youth in and improving health-related self-management skills and behaviors [17,19]. We scanned app stores for concussion-related apps and found that concussion-related apps exist, but they are primarily focused on concussion identification, diagnosis, and general information about concussion symptoms and recovery recommendations. These apps are not focused on concussion self-management, are not specific to pediatric concussion, do not emphasize strategies or the provision of tools to promote recovery, and do not allow tracking of personal information or recovery progress. Furthermore, most apps have not been validated in the peer-reviewed literature, and thus, their efficacy in helping youth manage concussion is unclear. Some efforts have been made, as indicated in the literature, to develop and evaluate apps for pediatric concussion management. For example, a study [20] evaluated the effects of a gamified mobile phone app in promoting health management in teenagers with persistent postconcussion symptoms, and it showed promising initial results for the use of mobile phone apps for the management of postconcussion syndrome [20]. However, this app focuses on improving the management of postconcussion syndrome that is experienced by only a subset of all concussed youth [21], and the app does not provide guidance throughout the entire recovery process, thus missing the opportunity to be preventative and guide youth from the onset of injury. SMART, another app developed for pediatric concussion management, is a Web-based educational and self-management program; its initial results show promise for the use of apps for pediatric concussion management [22-24]. However, a usability study of this app identified that some users felt the time and reading required to complete the program would be too difficult for children to comprehend and complete [22-24]. This may suggest the program requires a considerable amount of physical and cognitive effort to use, and the safety with which this app can be used by concussed youth is unclear and should be evaluated in addition to evaluating its efficacy. In addition, the app focuses on managing and tracking symptoms, instead of empowering or enabling the user to implement specific concussion management strategies; Zasler et al discussed that if symptoms persist, then focusing on symptoms might be counterproductive [25]. A technical limitation of Web-based apps is that they require an internet connection, which may not always be available or reliable, limiting access to individuals who have reliable access to the internet. In contrast to Web-based or HTML apps, native apps offer robust offline functionality, which is preferred for mHealth tools targeting individuals who may live in rural areas with poor internet connectivity or who do not have access to the internet [26,27]. Native apps also provide a richer user experience and better and more innovative capabilities than Web-based apps [26,27]. There is a need to develop and evaluate tools that are easily accessible to youth, guide them throughout their concussion recovery, educate them on and assist them in implementing best practice concussion management strategies, and that require minimal engagement by the user to ensure safety.

### Usability

Technologies with inadequate consideration of the needs of the intended users are difficult to learn, and these will be misused or underutilized and will ultimately fail to accomplish objectives originally set out [28]. Usability studies are commonly used to evaluate mHealth technologies [29-31], and they focus on measurable user performance and preference metrics. Usability is defined as the extent to which a product can be used by specified users to achieve specified goals with effectiveness, efficiency, and satisfaction in a specified context of use [32,33]. Having a user perform a set of tasks that relate to product features and are representative of the tasks that the user may use the technology for is an excellent way to determine the usability of a feature or feature workflow [34,35]. It is important to perform a usability study for an mHealth technology with prospective end users to effectively determine how well the target audience interacts and relates to a technology.

The objective of this research was to develop and evaluate the usability of a mobile phone app that will help enable youth to better self-manage concussion by providing easy access to expert-informed concussion management information and strategies and a tool that will guide youth in self-managing and tracking concussion recovery. Overall, this app is expected to improve the quality of life of youth who have experienced a concussion by providing recovery support to enable safe return to daily activities of meaning and importance (eg, school, family, social activities, and sport activities).

## Methods

### Overview of Application, Prototype Design, and Development

A user-centered design approach and Agile development methods were used to design and develop the *NeuroCare* mobile phone app to ensure that it was useful and usable for the end-user population. The design, development, and improvements to the prototype were carried out using an iterative and incremental development (IID) approach [36] with the support of the design team. The design team consisted of the key stakeholders and user proxies that included health care professionals, concussion experts, business personnel, and brain injury researchers who were iteratively involved in the design of this technology, that is, assisted in identifying the end users, creating a target user group profile, creating a persona, identifying design requirements and design principles, identifying mobile app’s features and functions, and identifying app content and design.

The design team identified that the primary end users of this technology are concussed youth aged between 10 and 18 years, and the secondary end users are the health care professionals who are involved in concussion assessment and management. Concussed youth can use this app to better self-manage their concussion through the implementation of evidence-informed concussion management strategies and progress tracking. Health care professionals can direct their youth clients/patients to the app and work with the youth to review concussion recovery progress and provide better direction and support. Both end users will use this app to improve communication between each other, which has shown to improve patient health outcomes, specifically emotional health and symptom resolution [37]. Youth may gain access to the app through Web-based app stores. The app can be used to support concussion recovery from the time of injury through to recovery and may be the most beneficial once the user begins to reintroduce daily activities at a gradual pace so that these do not provoke symptoms. Youth are instructed to use the app daily throughout their concussion recovery and share their progress (eg, how they have been feeling and the strategies implemented) with their health care professional to receive further support and feedback in managing their concussion (eg, advice and assistance regarding recommended and new strategies to implement). In addition, if health care professionals are aware of the app, they can direct youth to the mobile phone app upon concussion diagnosis and during recovery. As a result, both end-user groups were involved in testing the usability of this app.

The scientific content of this app, including the concussion management strategies (eg, energy conservation, sleep, nutrition, and relaxation strategies) and supplementary concussion management information found within this prototype was adapted from the *Concussion & You* program. Through IID of the app prototype, the design team identified opportunities to add to the current *Concussion & You* content, for example, the design team found that a new concussion management strategies section could be a beneficial addition for concussed youth. As a result, the design team developed a beta concussion management strategies section titled *Social Goals*, which includes strategies based on the current best practices and expert opinion to help reduce the impact of social isolation and depression that may accompany a concussion diagnosis [6,38,39]. The user interface (UI) of this prototype was initially influenced by the *Concussion & You* program strategy planning tool found in the program’s handbook [12], and this initial design was iteratively assessed by the design team using the IID approach, which resulted in the final UI that was used for the end-user usability study. For further information about the development process and design of the app see the dissertation *Development and Usability Testing of a Smartphone Technology for the Self-Management of Pediatric Concussion* [7].

**Figure 1:**
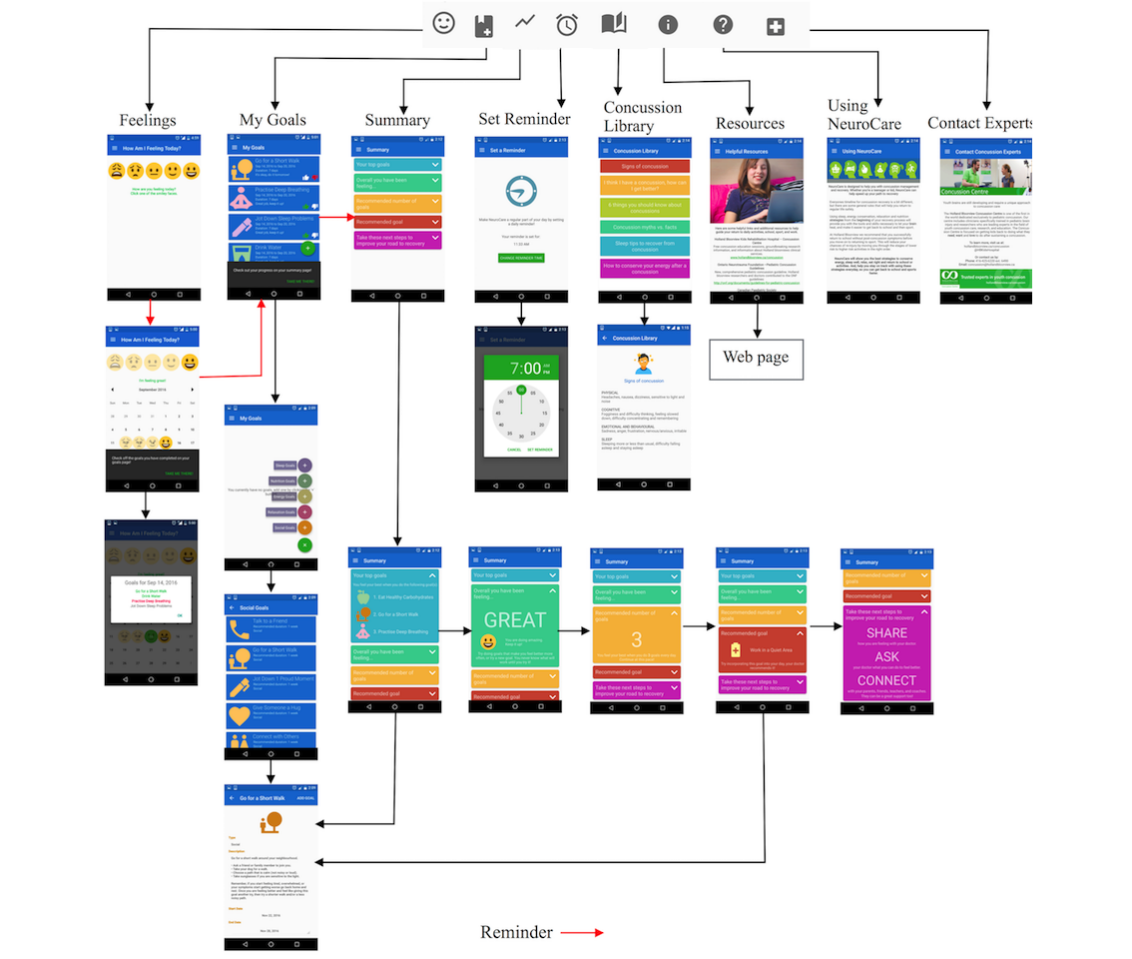
Final prototype information architecture. The app has a total of 8 main sections, with the Menu icons shown near the top. The arrows show how each screen is linked to the Menu, and how screens are linked to other screens within the application; the arrows indicate how a user could navigate through the different screens of the app.

The app’s information architecture is shown in Figure 1 (see Multimedia Appendix 1 for a higher resolution image); the 8 main sections of the application are divided into 2 distinct groups: destinations that aid self-management actively through action (ie, Feelings, My Goals, Summary, and Set Reminder pages) and destinations for aiding self-management passively through information (ie, Concussion Library, Resources, Using NeuroCare, and Contact Experts). The key features of the app are that it guides concussed youth in creating a personalized concussion recovery self-management plan, allows youth to track how they are feeling each day, provides daily reminders, and provides feedback and recommendations on how youth can improve their concussion recovery. A key design principle required that navigation through the app should be intuitive, simple, and demand minimal engagement from the concussed youth to ensure the app could be used safely (ie, to avoid symptom exacerbation). For example, an alternative path is indicated by the red arrows in Figure 1, which guides the user to complete the required daily actions using a short and concise workflow that requires minimal user engagement: these daily actions ask the user to select how they are feeling today on the *Feelings* page, go to the *My Goals* page, and select the competition status of each goal, and then navigate to the *Summary* page to check their progress. At the end of the week, youth are asked to review and revise their concussion recovery plan, which includes adding or removing goals based on guidance from their physician and the *Summary* page. The fully functional mobile phone prototype was further evaluated through this usability study with end users.

### Participants

A total of 14 participants were recruited for this study: 7 youth with a history of concussion and 7 health care professionals involved with concussion assessment, management, or research. Participants were excluded if they were younger than 10 years or older than 18 years; had not used mobile phone apps; were non-English speakers; were currently experiencing postconcussion symptoms; or if they had any physical, visual, or cognitive problems that may have precluded them from being able to use the mobile phone technology in the traditional way. Informed consent was obtained by all participants and/or their parents before participation. The study was approved by the Research Ethics Board at Holland Bloorview Kids Rehabilitation Hospital (REB#16-632) in Toronto, Canada.

### Protocol

We conducted a formative lab-based usability study [32,40] with the fully functional mobile phone prototype. Formative lab-based usability testing is a widely used usability testing approach that is iterative in nature [32]; the goal of this testing is to make improvements in design before releasing the product [32]. This includes identifying and diagnosing the problems, making and implementing recommendations, and then re-evaluating the product [32]. In formative usability studies, the most significant usability findings are observed with the first 5 participants [41,42]. This study was conducted at either Holland Bloorview Kids Rehabilitation Hospital or at the health care professional’s place of practice. The study was conducted in a quiet room and took 30 to 45 min to complete.

After the participant provided consent to participate in the study, they were asked to complete a demographics form. Then, the participant was introduced to the *think aloud* approach using a short video [43]. The *think aloud* approach asks the user to continuously verbalize their thoughts about their underlying thinking behind their interactions while using a technology by verbalizing what they are doing and why, stating when they encounter a problem, and how they feel while using the technology [43]. Next, the objective usability of mobile phone app was assessed by asking participants to complete 12 tasks (Table 1) using the mobile phone app while *thinking aloud*. During these tasks, the participants were audio-recorded, and a mobile phone screen recording app (AZ Screen Recorder [44]) was used to record the mobile phone screen; this included recording screen clicks and navigation to aid in identifying usability issues. The participant’s actions as well as performance metrics, such as task success, the time on task, and the number of errors and assists were also observed and documented using pen and paper notetaking. In addition, any issues the participants faced while using the technology, including issue type, frequency, and severity were documented. After the completion of all the tasks, the subjective usability and usefulness of the users were assessed using the System Usability Scale (SUS) [45], a posttest questionnaire, and an exit interview (described below).

**Table 1:** Usability study tasks list. The usability issues or technology features that each task attempted to investigate are listed followed by the task instructions.

Task	Features tested
1	Using the visual scale: enter the application, and answer the “how are you feeling today” question with OKAY using the visual scale.
2	Finding and adding a goal on the *My Goals* page: add a specific *Social* goal to the action plan for a duration of 1 week.
3	Setting a reminder: set a reminder 1 min from the current time.
4	Responding to the reminder notification: respond to the notification reminder that was set in Task 3.
5	Using the visual scale and using the prompt (ie, toast notification): answer the “how are you feeling today?” question with GOOD. Then, use the prompt to go from Home screen to the *My Goals* page.
6	Setting goal competition status and using the prompt (ie, toast notification): set the completion status for goals on the *My Goals* page, and use the prompt to go from the *My Goals* page to the *Summary* page.
7	Finding and adding the recommended goal: add the physician-recommended goal on the *Summary* page to action plan for a duration of 2 days.
8	Deleting a goal from the action plan: delete the *Social* goal originally added in Task 2.
9	Setting goal completion status and using the prompt (ie, toast notification): set the completion status for goals on the *My Goals* page, and use the prompt to go from the *My Goals* page to the *Summary* page.
10a	Finding concussion education information: find the *concussion myths versus facts* educational page.
10b	Navigating to the Home screen: return to the Home screen of the app.
11	Reviewing and comprehending feeling and action plan history: find out how you were feeling yesterday, and identify the completion status of yesterday’s goals.

#### Demographics Form

The demographics form for the 2 participant groups (youth and health care professionals) were customized for each group. Youths were asked questions regarding their concussion history and experience with managing a concussion, whereas health care professionals were asked about their involvement in concussion assessment, management, and/or research. For both participant groups, data on age, sex, and if they owned/had access to a mobile phone and/or tablet were collected. Furthermore, both groups were asked to answer questions about their perception of concussion knowledge and management in Canada using a 7-point Likert scale (1—strongly disagree, 7—strongly agree) and open-ended comments.

#### Usability and Usefulness

Usability is defined as the extent to which a product can be used by specified users to achieve specified goals with effectiveness, efficiency, and satisfaction in a specified context of use [32,33]. The objective usability of the prototype was evaluated by measuring the extent to which the prototype could be used to complete specified tasks with effectiveness and efficiency [32,33], and the subjective usability was evaluated by measuring satisfaction using the SUS, posttest questionnaire, and exit interview. Subjective/perceived usefulness was measured using the posttest questionnaire and exit interview.

##### Objective Usability: Task Performance (Observational Notes, and Audio and Screen Recordings)

###### Task Success and Number of Errors

The effectiveness of this prototype was evaluated by measuring the number of errors and assists and the number of tasks that were completed successfully (task success). Task success is the most widely used usability performance metric; if a user cannot complete a given task, then there is likely a problem with the technology [32,33,40]. Errors and assists also indicate effectiveness; both are useful in pointing out particularly confusing parts of a technology [32,33,40,46]. Errors were defined as any action that caused the participant to deviate from the path to successful task completion. Assists were defined as any assistance provided to the participant to aid in task completion; participants were provided assists only when they were having a considerable amount of difficulty with a task.

###### Time on Task

To measure the amount of effort (efficiency) with which participants completed each task, time on task was also measured, which included time for errors and corrections [32,33,40]; the faster the user completed a task, the lower the amount of effort required to complete a task, thereby offering an overall better experience. It is important to ensure that time is measured accurately and consistently [32,40,46]. We marked the start of each task as the time when the participant was told to start attempting the task, and the end time was marked as the time when the participant said “I am done”; waiting for the participant to say they are done is important, so that detectable usability issues do not go unidentified [32,40,46]. Although participants were asked to *think aloud* during this usability test, time-on-task data were still collected; however, using a concurrent *think aloud* protocol may impact task completion time. A solution is to ask participants to *hold* any longer comments until after a task is completed [32]; this solution was used to ensure task completion times were as accurate as possible while using the *think aloud* protocol. The number of, unnecessary actions or, actions exceeding the minimum number of actions required for a task is also indicative of the efficiency of a technology and were recorded [32,40,46]; it is possible for a task to have a fast completion time but still require a high amount of effort.

##### Subjective Usability and Usefulness: System Usability Scale, Posttest Questionnaire, and Exit Interview

Following the completion of the 12 tasks using the mobile phone app while *thinking aloud*, participants completed 2 posttest questionnaires, both focused on measuring the prototype’s usability; the SUS [45] was issued first followed by a more general posttest questionnaire focused on usability and usefulness [32,47].

The SUS consists of 10 questions and uses a 5-point Likert-scale answering scheme to get a reliable and robust evaluation of a product [32,45,48]. The SUS is a validated and reliable measure of the subjective or perceived usability of a system with small sample sizes (ie, 8-12 users) [32,45,49]. The SUS questionnaire was modified to be more youth-friendly (age-appropriate language) and customized for assessing a mobile phone app.

Furthermore, an additional and more general posttest questionnaire was used to measure the usability and usefulness, that is, “the degree to which a product enables a user to achieve his or her goals, and is an assessment of the user’s willingness to use the product at all” [50]. An adapted version of a usefulness questionnaire that was developed and validated by Davis was included as part of the posttest questionnaire to understand the perceived usefulness of this technology [50]. Additional questions were included to assess subjective satisfaction to complement the SUS score and better understand technology satisfaction. These questions were adapted from the Usefulness, Satisfaction, and Ease of Use questionnaire [32,51].

After completing the questionnaires, the participant was invited to take part in a semistructured exit interview; follow-up interviews are commonly used in usability studies where the researcher meets with the participants one-on-one to discuss in detail what the participant thinks about a specific topic in question, discuss usability issues, and clarify comments or behaviors exhibited during the usability study [43,52]. This exit interview was adapted from previous literature specific to usability evaluation [32,47,50,51,53], and it allowed the participant to share comments and opinions on the mobile phone technology, answer questions regarding the prototype’s usability and usefulness, discuss any problems they encountered while completing the assigned tasks, assist in clarifying and resolving usability issues, and to clarify key comments or behaviors exhibited during the *think aloud* protocol.

For this study, the primary outcome measures were the task success, time on task, errors, and SUS scores. The task success and errors per task evaluated the effectiveness, whereas time on task measured efficiency, and satisfaction was evaluated using the SUS, posttest questionnaire, and the exit interview. The secondary outcome measures were the usability issues, unnecessary actions, assists, and the usefulness of the app.

### Data Analysis

A triangulation approach [32,40,46] was used to identify the key usability issues with the mobile phone technology prototype. Descriptive statistics (eg, frequencies) were used to analyze all performance data (ie, task success, time on task, number of errors, unnecessary actions, and assists) and all close-ended demographic and posttest questionnaire data. Time-on-task data were analyzed using a measure of central tendency (ie, mean). Data from the think aloud protocol, exit interviews, screen recordings, and questionnaires for the tasks/questions indicating usability/usefulness issues were examined to identify the cause of, and the possible solutions for the issues using the approach described by Dumas and Redish [46]. The SUS questionnaire was analyzed using the procedure described by Brooke [45]; descriptive statistics (ie, measures of variability and central tendency) were also used to analyze the SUS scores and demographics data.

## Results

### Demographics

A total of 14 participants were recruited for this study: 7 youths with a history of concussion, and 7 health care professionals. Table 2 provides a summary of study participant demographics.

**Table 2:** Youth (n=7) and health care professional (n=7) demographics.

Participant group, category			Statistic
**Youth**			
	Age (years), mean (SD)		12.7 (1.9)
	Gender, female, n (%)		5 (71)
	Months since most recent concussion, mean (SD)		18.9 (4.7)
	Even with health care professionals helping me, I felt confused about what I should do to manage my concussion(s): agreed/strongly agreed, n (%)		5 (71)
	I either own, or have daily access to, a smartphone, n (%)		7 (100)
	I either own, or have daily access to, a tablet, n (%)		7 (100)
**Health care professionals**			
	Age (years), mean (SD)		42.9 (15.7)
	Gender, female, n (%)		7 (100)
	**Type of health care professional, n (%^a^)**		
		Neuropsychologists	2 (29)
		Occupational therapists	2 (29)
		School nurses	2 (29)
		Physical medicine and rehabilitation physician	1 (14)
	Years of work experience in this role, mean (SD)		9.6 (7.3)
	**I find all youth in Canada are given enough information to manage their concussion, n (%)**		
		Disagreed/strongly disagreed	6 (86)
		Slightly disagreed	1 (14)
	I find pediatric concussion is managed in a consistent and standardized manner by all health care professionals in Canada: disagreed/strongly disagreed, n (%)		7 (100)
	I either own, or have daily access to, a smartphone, n (%)		7 (100)
	I either own, or have daily access to, a tablet, n (%)		4 (57)

^a^The total of the percentages sums to more than 100% due to rounding.

### Objective Usability

#### Task Success

**Figure 2:**
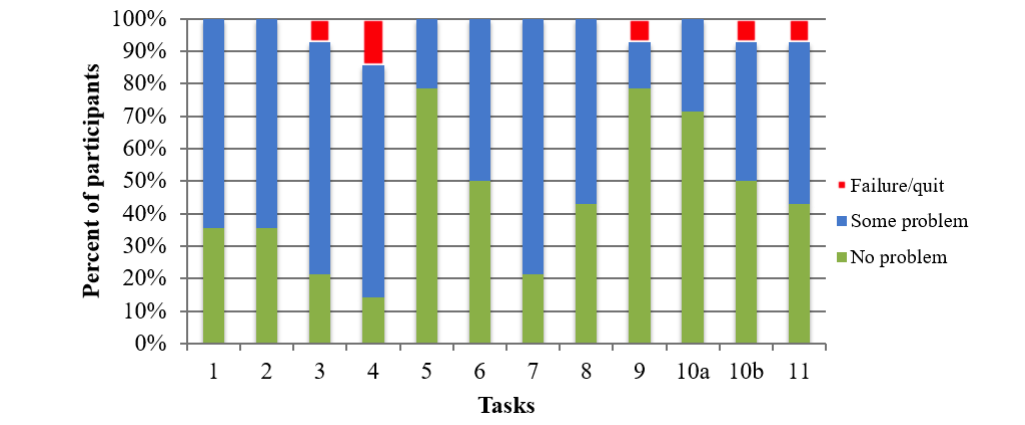
Percentage of participants by levels of task success per task. Task 1: select “how are you feeling today” using the visual scale; Task 2: add a *Social* goal to the action plan; Task 3: set a reminder; Task 4: respond to the reminder; Task 5: select “how are you feeling today” using the visual scale, and use the toast notification to navigate; Task 6: set completion status for goals, and use the toast notification to navigate; Task 7: find and add the recommended goal; Task 8: delete a goal; Task 9: set completion status for all goals, and use the toast notification to navigate; Task 10: (a) find concussion education information and (b) navigate to the Home screen; and Task 11: review and comprehend feeling and action plan history.

Mean task success rates were greater than 90% for 92% (11/12) of tasks, which indicates high usability. All participants successfully completed 7 of the 12 tasks (Table 1). A few participants were not able to successfully complete tasks 3, 4, 9, 10b, and 11. The percentage of participants who completed a task and their level of success (ie, zero problems [green], with 1 or more problems [blue], and task failure [red]) are shown in Figure 2.

#### Number of Errors

**Figure 3:**
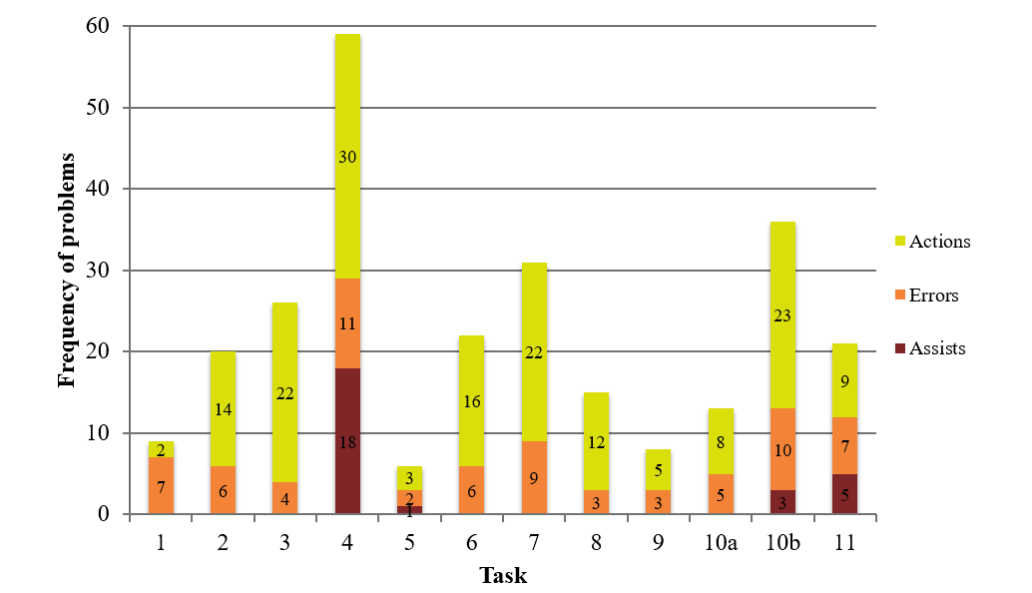
Frequency of assists, errors, and actions per task, across all 14 participants. Task 1: select “how are you feeling today” using the visual scale; Task 2: add a *Social* goal to the action plan; Task 3: set a reminder; Task 4: respond to the reminder; Task 5: select “how are you feeling today” using the visual scale, and use the toast notification to navigate; Task 6: set completion status for goals, and use the toast notification to navigate; Task 7: find and add the recommended goal; Task 8: delete a goal; Task 9: set completion status for all goals, and use the toast notification to navigate; Task 10: (a) find concussion education information and (b) navigate to the Home screen; and Task 11: review and comprehend feeling and action plan history.

The frequency of assists (red), errors (orange), and actions (yellow) for each task are shown in Figure 3, which shows that a number of assists were provided to participants for task 4, and some assists were provided for task 10b and task 11, demonstrating severe usability issues with task 4, and moderate usability issues with tasks 10b and 11. One task failure occurred for task 3 (Figure 2), for which 0 assists were provided and only a few errors occurred. However, many actions were taken that exceeded the minimum number of actions required for task 3; this may point to a minor usability issue. One failure also occurred for task 9 (Figure 2), but there were 0 assists required, only 3 errors, and 5 unnecessary actions across 14 participants, further indicating that task 9 may be a minor usability issue. Tasks 5 and 7 had a 100% success rate but still exhibited some issues. Only 1 assist was provided for task 5, and a miniscule number of errors and unnecessary actions occurred. Therefore, this task is not likely to point to a usability issue, but the cause of the assist was still investigated. However, task 7 resulted in 9 errors, and many unnecessary actions were taken; task 7 may point to a usability issue.

#### Time on Task

**Figure 4:**
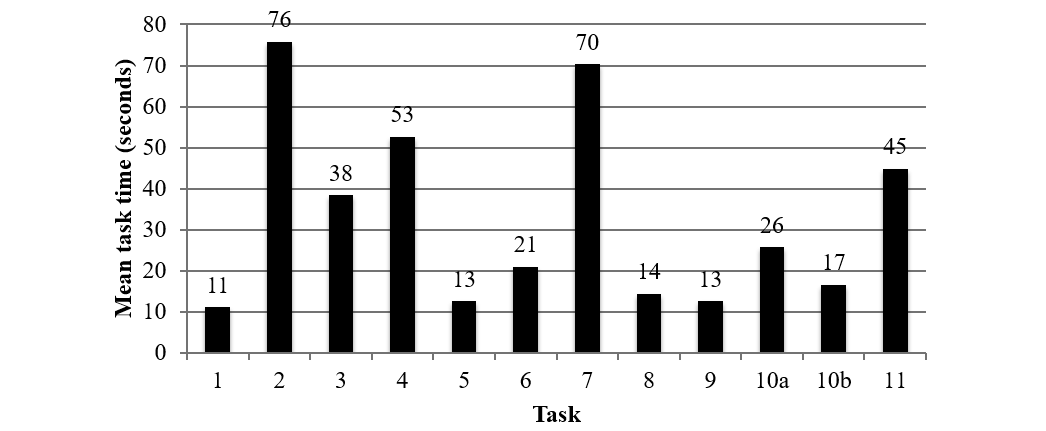
Mean task completion times per task, in seconds. Task 1: select “how are you feeling today” using the visual scale; Task 2: add a *Social* goal to the action plan; Task 3: set a reminder; Task 4: respond to the reminder; Task 5: select “how are you feeling today” using the visual scale, and use the toast notification to navigate; Task 6: set completion status for goals, and use the toast notification to navigate; Task 7: find and add the recommended goal; Task 8: delete a goal; Task 9: set completion status for all goals, and use the toast notification to navigate; Task 10: (a) find concussion education information and (b) navigate to the Home screen; and Task 11: review and comprehend feeling and action plan history.

The mean task completion time for each task is displayed in Figure 4. It was hypothesized that each task would take less than 2 min to complete; this hypothesis was confirmed. Figure 4 reveals that the mean time on task for each of the tasks was less than 77 seconds while using the *think aloud* protocol.

### Subjective Usability and Usefulness

#### System Usability Scale Questionnaire

Scores above 68 (SD 12.5) indicate above average usability [45,48,49]. The mean SUS score for this study was 81.9 (SD 11.3), indicating that, on average participants were highly satisfied with the usability of this mobile phone technology prototype. The mean SUS scores were calculated for the 2 groups, youth and health care professionals. The mean SUS score for youth was 87.5 (SD 8.5), whereas the mean SUS score for health care professionals was 76.4 (SD 11.5). SUS scores for 86% (6/7) of youth were equal to or above 82.5; 1 youth participant’s SUS score was 72.5. However, SUS scores for only 43% (3/7) of health care professionals were equal to or above 82.5. Furthermore, 2 health care professionals had scores of 65 and 60, which is considered below average. A small but significant correlation between age and SUS scores showing that SUS scores decrease as age increases has been shown in the literature [54], which may partially explain the lower SUS scores among health care professionals.

#### Posttest Questionnaire

In the posttest questionnaire, youth participants (n=7) were asked that in the hypothetical case they experience another concussion in the future, if they would use this app; 71% (5/7) strongly agreed that they would use this app, and the remaining participants agreed (n=1) and slightly agreed (n=1). When youth and health care professionals were asked if they would recommend this application to a concussed youth, 100% (n=7) of youth either slightly agreed or agreed (6/7 agreed and 1/7 slightly agreed), and 4 health care professionals agreed that they would recommend this technology to concussed youth. However, 3 health care professionals did not agree that they would recommend this technology; 1 health care professional neither agreed nor disagreed (ie, neutral), 1 slightly disagreed, and 1 disagreed. To better understand these 3 ratings, the open-ended responses, if available, were reviewed. The health care professional who neither agreed nor disagreed stated that they did not know enough about the app to recommend it. The health care professional who slightly disagreed was concerned that using the technology (ie, screen time) and the amount of reading/cognition involved may exacerbate symptoms. The health care professional who disagreed also mentioned that if a youth was to have to choose between spending allowed screen time on this app versus school work, they would recommend the functional task over the use of this app. A concern among some (2/7) health care professionals who did not agree or slightly agreed to recommend this app was that recommending this app meant they were recommending screen time to concussed youth; this was associated with the fear that extended screen time could lead to exacerbation of symptoms. However, most (4/7) health care professionals stated that they would recommend this app to concussed youth.

Health care professionals and youths were asked if this technology would be useful in helping the youth self-manage their concussion: 86% of participants either slightly agreed or agreed (9/14 agreed and 3/14 slightly agreed) that this technology would be useful in helping the youth self-manage their concussion, 1 youth neither agreed nor disagreed, and 1 health care professional disagreed. Analyzing the open-ended answers from the questionnaire revealed that the youth who neither agreed nor disagreed thought it would be hard for youth to remember to set goals and change how they feel every day; however, reviewing the task success and errors data revealed that this youth failed to complete task 3 (ie, setting a daily reminder). The health care professional who disagreed provided no explanation for their choice to disagree with the usefulness of this app. However, during the exit interview, this health care professional did mention that they believed the technology would be useful in helping youth self-manage their concussions if the technology tracked postconcussion symptoms and somehow tied symptoms with the goals.

## Discussion

### Principal Findings

This research described the development and evaluation of a mobile phone app to aid youth in self-managing concussion. A fully functional mobile phone app prototype was developed, and a usability study was completed to evaluate this technology. Usability issues with this technology were identified, and actionable recommendations were provided to resolve the issues; these issues should be resolved to improve the usability of the technology. Furthermore, some overarching issues, and corresponding recommendations to further improve the app are discussed.

As discussed, some tasks led to task failure, requiring assists, errors, and/or unnecessary actions. The tasks were analyzed, beginning with the tasks that indicated high severity issues, followed by low to medium severity issues [32,40,46]. The recommendations to improve the design of the mobile phone technology were developed and listed for high severity issues (Table 3) and low to medium severity issues (Multimedia Appendix 2).

**Table 3:** High severity usability issues and recommendations.

Feature	Problem	Recommendation
Reminders	Many participants had difficulty finding the reminder because they attributed a reminder to something that would pop-up in the middle of the mobile phone’s screen, emit a sound (*ringing*), and state that it is a reminder explicitly.	The reminder should explicitly state that it is the daily reminder that was set by the user from within the *NeuroCare* app, for example, the message within the reminder could state “Your daily reminder: How are you feeling today?”
Navigation menu	Some participants had difficulty locating/identifying the *main screen* of the app. Many participants attempted to click the *NeuroCare* brain logo in the navigation menu; participants thought that clicking this button should take them to the main screen. In addition, participants were attempting to look for a *home* icon to locate the main screen of the app.	The home screen of this technology is the “How Am I Feeling Today?” page. To ensure users can easily recognize that this is the main page, a *home* icon can be used to replace the current *smiley* icon for the “How Am I Feeling Today?” page. In addition, the brain logo in the navigation menu should be programmed such that when it is clicked, it takes the user to the main screen of the app.
Goals history	Participants were confused about the location of the goals history; the participants expected the goals history to be located on the *My Goals* page. However, when participants were asked if they thought it was useful to see their goals history in the *feelings calendar*, 100% (n=14) agreed.	The goals history should be moved to the *My Goals* page or the *Summary* page. Most participants expressed that they liked the calendar view, so it is recommended that the calendar format still be used to display the goal history.
Reminders (clock)	The current app clock-face was considered not intuitive and was too complex for participants, for example, some participants believed that the clock-face would only allow setting a reminder in 5-min intervals. Most participants mentioned that they identified more with a scrollable time picker and had difficulty using the clock-face time picker. One health care professional mentioned that the current clock-face might require a lot of cognitive effort.	To minimize the cognitive effort required to use this technology and improve usability, the current clock-face should be replaced with scrollable time picker.

In this study, the mean SUS score for this mobile phone app was found to be 81.9 (SD 11.3), which suggests that participants rated the app as highly usable; SUS scores above 68 (SD 12.5) indicate above average usability [45,48,49]. Sauro looked at the relationship between SUS scores and the *Net Promoter Score*. The latter asks individuals how likely they are to recommend a product to a friend or colleague [55]. Sauro found that individuals who rate a product with an SUS score of 82 (SD 5) tend to be *promoters* for the product [55]. Thus, the mean SUS score for this study of 81.9 suggests that people are likely to be *promoters* of this technology, and they are likely to recommend this technology to their friends or colleagues. More importantly, the high and consistent SUS scores provided by youth (mean 87.5, SD 8.5) suggest that they are more likely to be promoters of this technology than health care professionals (mean 76.4, SD 11.5). These results are in contrast to the SUS scores of a recently proposed intervention for pediatric concussion management titled SMART [24]. The SMART intervention was tested with 4 child/parent pairs, and the mean child SUS score was 81 (SD 22.8), whereas the mean parent score was 89 (SD 10.7) [24]. These scores suggest that the features and design of the SMART technology resonated better with older adults than children. In addition, the large SD in youth SUS scores indicates that some youth perceived the usability of the SMART technology as below average. In contrast, the results from our study suggest that all youth perceived the usability of the mobile phone technology as high and are likely to be promoters for this technology. Unlike the results for the SMART intervention, these results suggest that the features and design of this technology resonated better with youth than older adults. To better understand the discrepancy between SUS scores among health care professionals and youth, key responses to the posttest questionnaire were analyzed. In the posttest questionnaire, youth and health care professionals were asked if they would recommend this app to a concussed youth, 100% (n=7) of youth slightly agreed or agreed (6/7 agreed and 1/7 slightly agreed), and 4 health care professionals agreed that they would recommend this technology to concussed youth. However, 3 health care professionals did not agree that they would recommend this technology; 1 health care professional neither agreed nor disagreed (ie, neutral), 1 slightly disagreed, and 1 disagreed. These results support Sauro’s claim that individuals with SUS scores of 82 (SD 5) tend to be *promoters* for the product and are more likely to recommend the product. To better understand why some health care professionals did not completely agree to recommend the technology, the questionnaire’s open-ended responses, if available, were reviewed. A concern among some health care professionals (2/7) who did not agree or slightly agreed to recommend this app was that recommending this app meant they were recommending screen time to concussed youth; this was associated with the fear that extended screen time could lead to exacerbation of symptoms. This may be due to the fact that the best practice concussion management guidelines recommend a period of physical and cognitive rest following a concussion [56]. However, new international consensus has suggested a shorter rest period; now, the suggested rest period is of approximately 24 to 48 hours after injury versus the previously suggested rest period until resolution of postconcussion symptoms [57]. In addition, the benefits of the rest period have not been validated [6], and it is unclear whether physical and cognitive rest aid concussed youth in recovery [58]. In addition, findings from recent studies suggest that prolonged rest after concussion is associated with increased risk for the development of secondary problems [58-60]; these secondary problems include, anxiety/stress, physical deconditioning, irritability, social isolation, and depression [6,61]. Furthermore, it is unknown the extent to which youth adhere to the recommendations for physical and cognitive rest [62]. However, further development of this mobile technology should aim to demand even lower cognitive effort to ensure the technology can be safely used by concussed youth. Many steps can be taken to reduce the amount of cognitive effort required for youth to use this technology, for example, resolving the identified usability issues can reduce the amount of time, frequency of errors, and the amount effort that is required to use a technology; the usability issues can be resolved by applying the provided recommendations (Table 3 and Multimedia Appendix 2). In addition, future iterations of the app should inform users about symptoms that may be exacerbated when using a mobile phone app (eg, screen time may lead to increased headaches, fatigue, light sensitivity, and difficulty concentrating), provide methods to reduce possibility of symptom exacerbation (eg, inform users to decrease screen brightness), and notify users of what actions they can take if using the app leads to symptom exacerbation (eg, in the result of symptom exacerbation, stop engaging with the app, rest and attempt to re-engage when symptom exacerbation has resolved). Nevertheless, this usability study instructed participants to complete a series of tasks sequentially, which could have led to a high perceived cognitive workload, whereas concussed youth would only be expected to complete a subset of these tasks every day. Concussed youth would be expected to complete tasks 1 and 5 (Table 1) everyday; these tasks ask youth to enter the app, select how they are feeling, then go to their *My Goals* page, and state the completion status of the goals in their action plan. In addition, the youth can view their *Summary* page, which is also a part of task 5. According to the task completion times (Figure 4), on average these 2 tasks together required 24 seconds to complete. At the end of every week, youth would be asked to perform task 2 or task 7: these tasks ask youth to add a new goal. On average, these tasks take approximately 70 seconds to complete for each goal. Thus, youth would be expected to use this technology for less than 1 min on a daily basis and less than 2 min at the end of each week; this suggests that this technology requires lower effort per day compared with other concussion management interventions [12,24]. For example, during the usability study for the SMART intervention, analyzing the time-on-module data revealed that a mean of 49 min was spent on completing 6 of the 8 modules (2 modules were missing timing data), and there were a total of 103 webpages across the 8 modules [24]. To evaluate how safely this technology can be used by concussed youth and reduce health care professionals’ anxiety in recommending it, further work should include the analysis of perceived physical and cognitive workload as compared with other activities youth take part in during concussion recovery. A useful tool for evaluating perceived workload is the NASA Task Load Index questionnaire [63], which is a widely used and validated questionnaire [63] that can help to assess the perceived workload of this technology. In addition, further research is needed to trial this technology among a cohort of concussed youth to determine if the technology exacerbates postconcussion symptoms. Nevertheless, the majority (4/7) of health care professionals and all youth (7/7) stated that they would recommend this app to concussed youth.

In this study, health care professionals were asked about their perceptions of pediatric concussion management on the demographics form. When asked if they find all health care professionals in Canada manage pediatric concussion in a consistent manner, 100% (n=7) of the health care professionals disagreed or strongly disagreed with the statement. When health care professionals were asked if they find that all youth in Canada are given enough information to manage their concussion, 86% (6/7) of the health care professionals either disagreed or strongly disagreed with the statement; 1 health care professional slightly disagreed with the statement. These results are consistent with the current literature, which has shown that there is a lack of standardization and that significant gaps exist in the management of pediatric concussion in Canada [8-11]. The results from this study suggest that this technology may be useful in helping reduce the gaps in pediatric concussion management by providing easy access to expert-informed concussion management information and strategies and a tool that can guide youth in self-managing and tracking their concussion recovery.

All of the participants (n=14) in this study indicated that they either own or have daily access to mobile phone and tablet. This supports the findings from the recent Pew Internet & American Life Project that indicate that mobile phones have become the primary communication tool for the majority of adolescents in the United States [17,18]; 75% of those aged 12 to 17 years now own mobile phones [18]. Both youth and health care professionals have shown interest in this technology; as discussed earlier, 100% (n=7) of youth slightly agreed or agreed (6/7 agreed and 1/7 slightly agreed) and 4 health care professionals agreed that they would recommend this technology to concussed youth. In addition, 100% (n=7) of the youth participants agreed that they would use this technology if they were to suffer another concussion in the future, and 86% of participants either slightly agreed or agreed (9/14 agreed and 3/14 slightly agreed) that this technology would be useful in helping the youth self-manage their concussion. This suggests that this mobile phone app may be an accessible, useful, and a feasible concussion management intervention for concussed youth.

This usability study provided valuable end-user feedback from both youth and health care professionals. A number of usability issues were identified, and the corresponding recommendations to improve the design of the app were provided (Table 3 and Multimedia Appendix 2); many low and moderate severity issues were identified, and 4 high severity issues were identified. In addition, some recommendations to improve the safety, uptake, and overall design of the technology were provided. Our findings suggest that participants rated this mobile phone app as having high subjective usability as indicated by a mean SUS score of 81.9 (SD 11.3). In addition, mean task success rates were greater than 90% for 92% (11/12) of tasks, and most (11/12; 92%) tasks had a total error frequency of less than 11, which also suggests high objective usability. On average, each task was completed in less than 2 min, which suggests this app is highly efficient. The results of the posttest questionnaires suggest that youth and health care professionals are open to using this app for self-management of concussion in youth and feel that this technology would be useful in helping the youth in managing their concussions. Overall, the results from the study suggest that participants rated this technology as usable, acceptable to users, and that it may be useful in helping youth self-manage concussion. Further work should include the analysis of perceived physical and cognitive workload to evaluate the safely of this technology, applying the recommendations to resolve the identified usability issues, modifying features to reduce physical and cognitive workload, and conducting a second usability study. In addition, further research is needed to trial this technology among a cohort of concussed youth to evaluate the effectiveness and safety of this technology.

### Limitations

Most participants (12/14) were from Toronto, Ontario, and the participants who chose to take part in this study may have been more motivated, knowledgeable of concussion management, and comfortable with using mobile phones. Thus, this sample may not be representative of the general concussed youth and health care professional populations. We were unable to gain insight into how different age groups among children and youth may engage and rate usability differently as this would require a larger sample size with representation across ages. The data from usability study were analyzed and interpreted to identify usability issues. This could have biased the study results by not having interpreted a participant’s comments appropriately [32,49]. However, to reduce this bias, we confirmed all findings during the exit interview and used the recordings to enhance and clarify the findings. This study was conducted with the researcher present in the room; a limitation of this type of study is that the behaviors and performance of participants may be altered as a result of their awareness of being observed [64]. Although the results of this study suggest that the participants’ response to this mobile phone app has been very positive, further research is needed to trial this technology among a cohort of concussed youth to evaluate the effectiveness and safety of this technology and to identify the subpopulations for whom this intervention would be most effective.

### Conclusions

This research describes the development and usability evaluation of an innovative and accessible pediatric concussion management intervention in the form of a fully functional Android mobile phone app prototype *NeuroCare*. The results from our usability study indicate that participants rated this technology as usable, acceptable to youth and health care professionals, and that it may be useful in helping youth self-manage concussion. Consistent with the current literature, results from this research suggest that there are large gaps in the way concussion is managed from both the youths’ and health care professional’s perspectives. This technology is expected to help bridge the gaps in pediatric concussion management by enabling and empowering youth to self-manage concussion by providing easy access to expert-informed concussion management strategies and helping guide youth in managing and tracking their concussion recovery. Next steps should include resolving the identified usability issues, modifying features to reduce cognitive and physical workload, and then conducting another usability study that should include the evaluation of perceived physical and cognitive workload. Future work should trial this technology among a cohort of concussed youth to determine the effectiveness and safety of this technology as a concussion self-management tool/intervention.

## Appendix

Multimedia Appendix 1 Final prototype information architecture (higher resolution image). The app has a total of 8 main sections, with the Menu icons shown near the top. The arrows show how each screen is linked to the Menu, and how screens are linked to other screens within the application; the arrows indicate how a user could navigate through the different screens of the app.

Multimedia Appendix 2 Low to medium severity usability issues and recommendations.

## Abbreviations

CIHR: Canadian Institutes of Health Research
IID: iterative and incremental development
mHealth: mobile health
SUS: System Usability Scale
UI: user interface
