# Supplementary material for: A Mobile Phone App for the Self-Management of Pediatric Concussion: Development and Usability Testing
Source: JMIR Hum Factors. 2019 May 31;6(2):e12135. doi: 10.2196/12135 (PMC6658289; doi:10.2196/12135)
Supplement: Multimedia Appendix 1 [file humanfactors_v6i2e12135_app1.pdf]

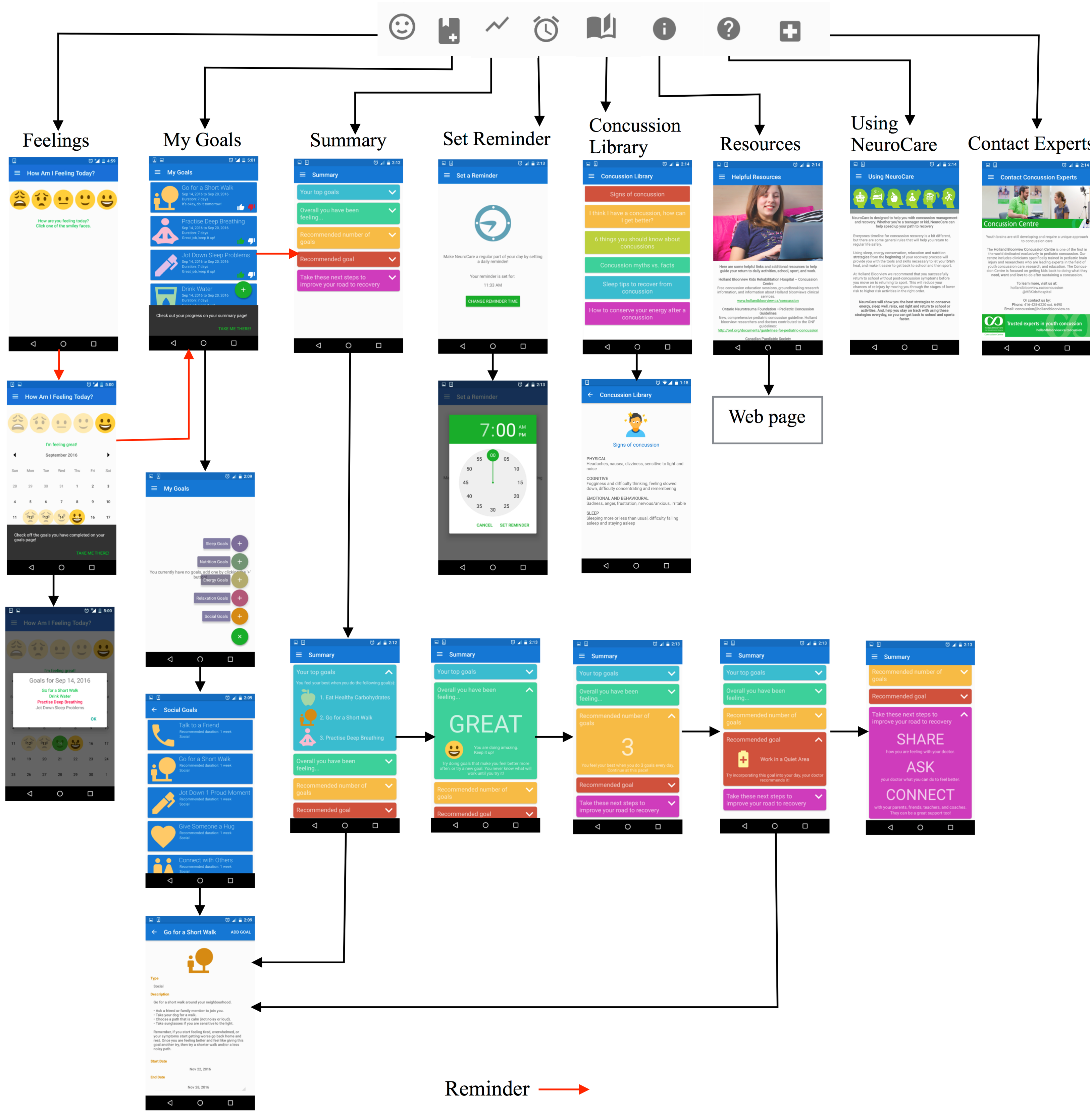

Feelings
My Goals
Summary
Set Reminder
Concussion Library
Resources
Using NeuroCare
Contact Experts
How Am I Feeling Today?
How are you feeling today? Click one of the smiley faces.
I'm feeling great!
September 2016
Check off the goals you have completed on your goals page!
TAKE ME THERE!
Goals for Sep 14, 2016
Go for a Short Walk
Drink Water
Practise Deep Breathing
Jot Down Sleep Problems
OK
Practise Deep Breathing
Jot Down Sleep Problems
Drink Water
Check out your progress on your summary page!
Sleep Goals
Nutrition Goals
Energy Goals
Relaxation Goals
Social Goals
Talk to a Friend
Go for a Short Walk
Jot Down 1 Proud Moment
Give Someone a Hug
Connect with Others
ADD GOAL
Your top goals
Overall you have been feeling...
Recommended number of goals
Recommended goal
Take these next steps to improve your road to recovery
1. Eat Healthy Carbohydrates
2. Go for a Short Walk
3. Practise Deep Breathing
GREAT
3
Work in a Quiet Area
SHARE
ASK
CONNECT
Set a Reminder
CHANGE REMINDER TIME
7:00
CANCEL
SET REMINDER
Concussion Library
Signs of concussion
I think I have a concussion, how can I get better?
6 things you should know about concussions
Concussion myths vs. facts
Sleep tips to recover from concussion
How to conserve your energy after a concussion
PHYSICAL
COGNITIVE
EMOTIONAL AND BEHAVIOURAL
SLEEP
Helpful Resources
Web page
Using NeuroCare
Contact Concussion Experts
Concussion Centre
Trusted experts in youth concussion
Reminder
