# Supplementary material for: A Mobile Phone App for the Self-Management of Pediatric Concussion: Development and Usability Testing
Source: JMIR Hum Factors. 2019 May 31;6(2):e12135. doi: 10.2196/12135 (PMC6658289; doi:10.2196/12135)
Supplement: Multimedia Appendix 2 [file humanfactors_v6i2e12135_app2.pdf]

Feature	Problem	Recommendation
Recommended goal	There is no *ADD* button in the recommended goal's section, on the Summary page. Some participants said they were unsure if clicking on the goal would add the goal, even though it did.	Make it clear that the goal can be added directly from this recommended goal section. One recommendation would be to add text beside the goal that says *ADD GOAL*.
Goal Completion Icons	The thumbs-up (complete) and thumbs-down (not complete) icons for each goal were confusing to some participants. Some participants thought these icons were used to indicate whether the participant liked or disliked a goal.	Change these icons to other icons, such as a checkmark (complete) and cross (not complete).<br><br>Another option is replace the current icons with a box that can be checked off.
Adding a goal	When adding a goal to their action plan, participants had to set the duration of the goal and state how they would accomplish it. Participants thought that after stating how it would be accomplished, and clicking the checkmark on the keyboard, the goal would be automatically added.	The button to add the goal was called *ADD GOAL* and was located in the top-right of the page. Changing the location of this *ADD GOAL* button to the bottom of the screen, underneath the "how the user will accomplish the goal" section, may avoid errors and improve the efficiency with which a user is able to locate the button.
Deleting a goal	When asked to delete a goal, participants began trying to *swipe left* a goal on the *My Goals* page thinking this would delete the goal.	Swiping elements to the left is a common UI tactic, to indicate item deletion or archiving. This should be implemented in this application as well.
Menu Icon	One healthcare professional did not recognize the menu icon and therefore did not instinctively use it to navigate throughout the application.	The *hamburger* icon for a menu is a universal design element in mobile applications. To mitigate a lack of knowledge in icon recognition and of the application's features, a short tutorial on how to use the application should be developed and included as an introductory video played when the application is first downloaded.
Visual Scale for Feelings	Cognitively, it may be hard to figure out which *smiley* corresponds to which type of feeling.	Add text underneath each smiley icon, indicating what each one represents.
